# Equine Nasal Polypi, with Profuse Discharge

**Published:** 1897-10

**Authors:** 


EQUINE NASAL POLYPI, WITH PROFUSE DISCHARGE.—This subject had an enlargement of the submaxillary lymphatics, and at the same time large quantities of mucus were passing from the nose. It was suspected that the nasal sinuses were filled with pus. The animal was trephined in the left frontal sinus, and large quantities of pus escaped. This treatment did not prove successful. The superior submaxillary sinus was then opened, and large quantities of pus escaped from that, but still the animal continued to discharge from the nostrils. Mallein was tried in two instances at intervals of two months, but did not produce any pointed reaction. The animal finally developed a large tumor, was declared incurable, and killed. Autopsy: Large masses of pus were found. The membrane and mucous membrane were extremely friable and very vascular. Ossified masses were found in some parts of the right superior maxillary sinus. On the back of the ethmoidal sinus there was found a large tumor, red in color, gelatinous and irregular shaped, and soft and friable. The pedicle of this myxoma was attached by numerous ramifications, and the periosteum of the other organs were healthy. —*Recueil de Médecine et Vétérinaire.*

